# Biomechanics of Traumatic Head and Neck Injuries on Women: A State-of-the-Art Review and Future Directions

**DOI:** 10.3390/biology12010083

**Published:** 2023-01-04

**Authors:** Gustavo P. Carmo, Jeroen Grigioni, Fábio A. O. Fernandes, Ricardo J. Alves de Sousa

**Affiliations:** 1 Centre for Mechanical Technology and Automation (TEMA), Department of Mechanical Engineering, Campus Universitário de Santiago, University of Aveiro, 3810-193 Aveiro, Portugal; 2 LASI—Intelligent Systems Associate Laboratory, 4800-058 Guimaraes, Portugal

**Keywords:** sex differences, biomechanics, brain injury, neck injury, finite element models, traumatic injury, chronic traumatic encephalopathy

## Abstract

**Simple Summary:**

With this review, the authors aim at providing the reader a concise biological and biomechanical description of the main contributions in the field of traumatic brain injuries and neurodegenerative outcomes for women, especially related to chronic traumatic encephalopathy. A review on numerical models created to address these issues is also performed, discussing the use (or the lack of use) of sex-specific validation experiments to validate those models. A discussion is also performed to alert to some considerations to be taken in account when numerically modelling those same injury scenarios.

**Abstract:**

The biomechanics of traumatic injuries of the human body as a consequence of road crashes, falling, contact sports, and military environments have been studied for decades. In particular, traumatic brain injury (TBI), the so-called “silent epidemic”, is the traumatic insult responsible for the greatest percentage of death and disability, justifying the relevance of this research topic. Despite its great importance, only recently have research groups started to seriously consider the sex differences regarding the morphology and physiology of women, which differs from men and may result in a specific outcome for a given traumatic event. This work aims to provide a summary of the contributions given in this field so far, from clinical reports to numerical models, covering not only the direct injuries from inertial loading scenarios but also the role sex plays in the conditions that precede an accident, and post-traumatic events, with an emphasis on neuroendocrine dysfunctions and chronic traumatic encephalopathy. A review on finite element head models and finite element neck models for the study of specific traumatic events is also performed, discussing whether sex was a factor in validating them. Based on the information collected, improvement perspectives and future directions are discussed.

## 1. Introduction

The human head is, without question, an essential body part. The skull encapsulates and protects the brain, which encompasses the central nervous system, responsible for controlling all other organs and, thus, necessary to sustain life. The intricacy and fragility of the brain require extensive research and mapping mechanisms in brain injuries.

Head injuries are one of the leading causes of death in the world. Over the years, the scientific community joined efforts to understand the biomechanics of these traumas and the best way to diagnose and prevent them. Traumatic Brain Injuries (TBIs) contribute to worldwide death and disability more than any other traumatic insult [1]. TBI is a broad concept that describes a vast dispersion of injuries that happen to the brain structures. The inflicted damage can either be focal (confined to one area of the brain) or diffuse (occurs in several brain areas). The severity of a TBI can range from a mild concussion to a severe injury that may result in death [2].

Despite the urgent research on this topic, literature studies of numerically-modelled TBI and concussion usually relate to male or unspecified-sex subjects. There are currently a growing number of studies evaluating factors associated with TBI; however, there remains relatively little research on women or sex differences [3].

When referring to Head and Neck (HN) injuries, one must consider the occurrence rate of each specific trauma in males and females. Concerning head injuries, male subjects are more likely to have extreme sport-related TBI [4,5], TBI resulting from road accidents [6] or, for example, in 2013, TBI-related Emergency Department visits, Hospitalizations, and Deaths (TBI-EDHDs) due to the following: being struck against or by an object; motor vehicle crashes; intentional self-harm; and assault [7]. However, women scored a higher number of TBI-EDHD as a result of falls, usually sustained by older adults.

Another head injury issue affecting mainly women is Intimate Partner Violence-related TBI (IPV-TBI), it being estimated that around one-third of women have experienced IPV at least once in their lifetime and that 23.2% of women have experienced severe physical violence by a partner, following a 2017 Centers for Disease Control and Prevention (CDC) survey [8]. The CDC defines IPV as physical violence, sexual violence, stalking or psychological aggression by a current or former intimate partner to both current and former spouses and dating partners [9]. IPV often includes physical assault (including injuries to the head and strangulation injuries). St. Ivany et al. [10] found a prevalence of 60% to 92% of abused women suffering a TBI correlated with IPV. A significant issue concerning IPV, sports-related impacts, or even falls are isolated or repetitive mild Traumatic Brain Injuries (mTBI) which, until recently, were often overlooked by health specialists and were rarely associated with hospitalization of the patient [11]. More recently, mTBI effects and long-term sequelae are being extensively studied, such as noise sensitivity [12], insomnia [13], cognitive impairment [14], visual field defects [15], changes in White Matter (WM) Fractional Anisotropy (FA) [16], among many others. However, most of these studies regarding mTBI lack sex-specific data, even though distinct effects on women and men are often reported in the literature [17,18].

Gupte et al. [19] performed an extensive literature review on sex differences in TBI, concluding that human studies are usually associated with worse outcomes in women than men, also showing that multiple factors including severity, sample size and experimental injury modelling may deferentially interact with sex to affect TBI outcomes.

Regarding neck injuries, these can be related to different types of whiplash (usually associated with rear-end vehicle collisions), neck fractures and cervical spinal cord injuries. Regarding Whiplash-associated Disorders (WAD), it is well established that its prevalence is higher in females when compared to males, usually more than double [20,21,22].

The following sections will discuss several neck and head injuries and how they are distinguished in terms of sexes in the literature. Then, the way such injuries are being modelled numerically will be addressed. A recap and future directions close the manuscript.

## 2. A Brief Recap on Brain and Neck Injuries

The brain is the most complex organ in the human body and is surrounded by a bone structure. One of the primary purposes of the skull bones is to protect and encapsulate the brain. The brain and the spinal cord make up the central nervous system. To control the body’s activities, it processes, integrates and coordinates the information received from the sensory organs. The human brain can be divided into three distinguishable entities: the cerebrum, the brainstem and the cerebellum [23,24].

The cerebrum consists of the left and right cerebral hemispheres. While the left and right hemispheres are similar in structure and function, some differences exist. Each of the hemispheres comprise inner white matter and outer grey matter tissue. White matter compartments consist mainly of myelinated axons, although it also contains unmyelinated axons. Grey matter, on the other hand, consists of a few cell bodies and mostly unmyelinated axons, dendrites, and glia cell processes, forming a synaptically dense region. The corpus callosum (CC) is a broad band of white matter carrying axons which connect the cerebral hemispheres [23,24,25].

The cerebrum is connected to the spinal cord by the brainstem. The brainstem consists of the pons, the midbrain, and the medulla oblongata. It has the critical role of regulating visceral organs [23,24].

The cerebellum plays a vital role in motor control. It is part of the metencephalon and serves as a control body for coordinating and fine-tuning movement sequences [26].

### 2.1. Microtubules Role in Axons

Brain cells include supportive glial cells and neurons. Brain functions are possible due to interconnections of neurons and the release of neurotransmitters in response to nerve impulses. Neurons consist of a cell body, axon, and dendrites. The transmission of information starts when a dendrite receives data in the form of signals from the axon terminals of another neuron. This transmission of information can cause the neuron to initiate an action potential. The action potential is transmitted along the neuron’s axon to the axon terminal to communicate with the cell body or dendrites of another neuron [27]. When an action potential reaches the presynaptic terminal, it triggers the release of a neurotransmitter into the synaptic gap that propagates a signal that acts on the postsynaptic cell [28].

Microtubules, neurofilaments and microfilaments form the axonal cytoskeleton [29]. Microtubules are the most robust cytoskeletal filaments in eukaryotic cells. Therefore, they play a significant role in various cellular processes. In neurons, they maintain structural stability and provide highways for axonal transport. Microtubules are stabilized and cross-linked to form the axonal cytoskeleton via microtubule-associated proteins [30]. Tau is an abbreviated term for the Microtubule-Associated Protein Tau (MAPT). There are six major tau isoforms due to alternative mRNA splicing. By binding to microtubules, tau loses its natively disordered state and contributes to essential structural and regulatory cellular functions. Moreover, within individual microtubules, tau controls microtubule polymerization, regulates axonal transport and controls microtubule structure [31,32]. Within the axon, tau promotes the packing of microtubules into well-organized, evenly spaced bundles [33]. To this day, two hypotheses have emerged to explain the packing of microtubules within the axon: the cross-bridging and the polymer brush hypothesis [34]. Despite its importance for axonal structure and function, the precise mechanism by which tau regulates microtubules packing remains poorly understood [30]. The addition of a site-specific phosphate group, also known as phosphorylation, is the primary mechanism for regulating tau activity [35]. Under physiological conditions, tau phosphorylation promotes the association with tubulin and stabilizes microtubule structure [30].

### 2.2. Chronic Traumatic Encephalopathy, CTE

Damage to the brain caused by external mechanical forces to the head is defined as a TBI. Damage to the brain structure occurs when a load exceeds the tolerance level of a brain tissue [36]. According to Graham et al. [37] TBI can be classified as focal and diffuse. Focal injuries include contusions, intracerebral hematomas, lacerations of the brain and burst lobe lesions. Typical diffuse brain injuries include Diffuse Axonal Injury (DAI), hypoxic brain injury, brain swelling, and diffuse vascular injury [37]. Bigler et al. classified post-traumatic neuropathological changes into primary and secondary changes. Primary changes consist of immediate alterations after a TBI, whereas secondary post-traumatic changes can include complex vascular and neuroinflammatory mechanisms.

Even though TBI is a leading cause of worldwide death and disability, sex differences in the pathophysiology and recovery are poorly understood, limiting clinical care and successful drug development [19]. Recent studies have convincingly documented a close correlation between TBI and pituitary dysfunction [38] and CTE [36]. Currently, the only method to reliably diagnose CTE is post-mortem histopathology with a complete autopsy and immunohistochemical analysis [39]. Although the exact cause of mechanically induced tauopathy is unknown, CTE is usually associated with repeated mTBI, not a single trauma [40], although there are also reports that suggest a single moderate-severe TBI can induce CTE-like pathologies [41,42]. At this time, it remains controversial whether misfolding of tau into Neurofibrillary Tangles (NFTs) is a consequence or a cause of neurodegeneration [43]. An accumulation of hyperphosphorylated tau (p-tau) protein, progressive axonal failure, and gradual structural degradation are the hallmarks of the disease [44].

To understand sex differences in the pathologies following HN injuries, it is vital to establish where the conditions occur and define the respective neuroanatomical and hormonal differences according to sex [45]. The further discovery of the sexual dimorphism of the brain will lead to important insights regarding the neurodegenerative disorders and their different ages of onset, prevalence, and symptomatology between males and females.

### 2.3. Axonal Injury

According to Braun et al. [40], mechanical stretching of axons impairs axonal transport by disrupting the organization of microtubules.

Under physiological strain and strain rates, tau-microtubule interactions deform axons reversibly and make them an almost entirely elastic material [46]. Under abnormal conditions, tau-microtubule dynamics result in brittle axons at pathological strain and strain rates, and their cytoskeleton becomes more easily damaged [47]. Cytoskeletal destruction disrupts axonal transport; the transport products build up at the site of damage, the axon starts to swell, and will eventually break [48]. Upon retraction of the transected axon, a bulb forms close to the cell body, which is a classical hallmark of DAI [49]. Recent findings suggest that axonal failure is a gradual interplay of biomechanical and biochemical events, including the initial biomechanical injury followed by secondary biochemical events within hours or days, a phenomenon known as the secondary axotomy [50].

The disruption of microtubules is believed to precede the detachment of tau proteins from microtubules and subsequent tau hyperphosphorylation. Therefore, it is likely that microtubule disruption caused by axonal injury may impair presynaptic function through tau-independent mechanisms. In contrast, tau hyperphosphorylation is essential for aberrant accumulation of tau proteins in postsynaptic structures and subsequent postsynaptic dysfunction [40].

### 2.4. Molecular Mechanism of CTE and Tau Pathology

The tau-microtubule compound plays a significant role in regulating axonal cytoskeletal structure, mechanics, and function [51].

Recent studies [40,47] have shown direct evidence that cell-scale mechanical deformation can lead to tauopathy and, therefore, to synaptic deficits in neurons. It was shown that the mechanical energy of TBI alone could induce tau hyperphosphorylation and mislocalization. Yet, the precise mechanisms of tau-mediated neurotoxicity are still not completely understood. Several pathological mechanisms are currently being studied.

According to recent studies [36,40,47] p-tau translocates to the cell body and aggregates to form NFTs, leading to impaired axonal function. Under physiological conditions, tau is an intrinsically disordered protein before an array of posttranslational modifications [52].

Furthermore, pathological hyperphosphorylation reduces tau to microtubules binding affinity, promotes tau fibrillization, and disrupts intracellular function [53]. In addition, there is growing evidence that tau aggregates can recruit other tau aggregates and then spread to surrounding regions. Eventually, intracellular transport is disrupted, which induces synapse loss, cell death, and loss of neural circuits. Ultimately, neurodegeneration leads to cognitive decline and impaired motor function. Pathologies that share these common neurodegenerative pathways are defined as “tauopathies” [30].

### 2.5. Location of Tauopathies

Although NFTs are a common pathophysiological hallmark of tauopathies, their distribution and spreading throughout the brain differ between diseases. Studies have shown that white matter tissue exhibits a gradual stiffness gradient [54] and a discrete stiffness jump across the grey-and-white-matter interface [55]. In addition, simulations revealed stress discontinuities at the tissue-vasculature interface [56].

DAI occur primarily at the grey-and-white-matter and tissue-vasculature interface, where mechanical stress fields undergo a discrete jump, supporting the concept that biomechanical factors initiate CTE [50].

In CTE, p-tau NFTs primarily aggregate focally and perivascularly in the cerebral cortex, with a predilection for deep sulci in the superficial neocortical layers [36]. These areas of NFT aggregations correlate with the brain regions that experience the most considerable strains during impact [56]. During an impact, a finite element model by Braun et al. [40] predicted that the first principal strains were most significant in the sulcal depths and that the most considerable strains were stretch strains. In addition, a depression model demonstrated that the most considerable strains occur immediately around the vessel. Recent studies have shown that CTE-associated p-tau NFTs accumulate in regions of the brain that undergo the most significant mechanical deformation during TBI [40]. Eventually, NFTs spread prion-like to the neocortex, medial temporal lobe, diencephalon, basal ganglia, and brainstem [36]. The gradual loss of neurons across the brain leads to pronounced grey and white matter atrophy, enlarged lateral and third ventricles, cavum septum pellucidum, septal fenestrations, locus ceruleus and substantia nigra depigmentation, thalamic and hypothalamic atrophy (including the mamillary bodies), as well as an overall reduction in brain mass [57,58].

In contrast to CTE, in Alzheimer’s Disease (AD), the NFT localization is more uniformly distributed in the deeper-lying cortical layers and not concentrated in sulcal depths, or perivasculature [59]. These results support the hypothesis of a causal relationship between mechanical deformation and tau pathology in CTE patients.

### 2.6. Pituitary Dysfunction

TBI can lead to varying degrees of Post-Traumatic Hypopituitarism (PTHP). The most common hormonal deficits after TBI include decreased Growth Hormone (GH) secretion and hypogonadism, followed by hypothyroidism, hypocortisolism, and diabetes insipidus [60,61]. In a study by Schneider et al. [62], the incidence of PTHP after TBI was evaluated. Three months after experiencing head trauma, it was shown that in 22 patients with mild, moderate, or severe TBI, 36.4% of patients showed subnormal responses in at least one hormonal axis.

The pituitary gland is uniquely situated within a protective bone structure called sella turcica and is attached to the brain by blood vessels and neurites. There are several cell types within the pituitary glands that produce various hormones; they regulate the endocrine activities of the adrenal cortex, thyroid, and gonads. It can be divided into the larger anterior pituitary (adenohypophysis) and the smaller posterior pituitary (neurohypophysis). The vasculature of the gland is a complex system of blood vessels which connects the adenohypophysis to the hypothalamus. The blood vessels carry the hypothalamic releasing and inhibiting hormones that control the pituitary hormone-producing cells. About 70–90% of the blood is supplied by the long portal vessels [63].

Although the exact underlying pathogenesis of PTHP has not yet been elucidated, various theories have been studied. A widely-accepted hypothesis suggests that as a consequence of a TBI, there is an ischemic insult to the pituitary gland [64]. The long hypophysial vessels are in particular vulnerable to vascular injury. The lateral somatotroph and gonadotroph axes directly depend on the long portal vessels. The hormone deficiencies pattern of hormonal loss and cellular distribution, which frequently involve the lateral somatotroph and gonadotroph axes, supports the vascular hypothesis [65]. In addition to ischemic injury, various other possible underlying pathophysiological pathways exist. Two other underlying mechanisms of impairment in the anterior pituitary have been extensively studied, neuroendocrine insults to the pituitary gland [66] and hypothalamic-pituitary autoimmunity mechanism [67].

## 3. The Particularities of HN Injuries for the Female Population

### 3.1. Sex Differences in Injury Outcome

The role of sex in the outcome after TBI remains controversial. On the one hand, multiple clinical studies have shown more favourable outcomes in women than men [68,69,70,71], other studies demonstrate no significant sex effect [72,73,74,75,76,77,78,79,80,81] or more favourable outcomes in men compared to women [82,83,84,85,86,87,88]. According to a recent scoping review by Gupte et al. [19], the largest fraction (47%) of the 156 studies reported less favourable outcomes in women than men, 26% found better outcomes in women, 18% found no sex difference and only about 9% reported mixed results, with women performing better on certain outcome measures and men on others. Within larger studies (>10,000 patients), less favourable outcomes were reported for women. Since larger sample sizes imply greater statistical significance, these large studies could be more reliable predictors of sex differences in TBI outcomes.

### 3.2. Neuroanatomical Sex Differences

Ruigrok et al. [45] showed in a meta-analysis that across a wide age range, from newborns to individuals over 80 years old, differences in overall brain volumes are sustained between males and females. On average, males have around 8–15% larger total brain volumes as well as a higher Intracranial Volume (ICV), higher tissue/region-specific volume [45], a more significant overall amount of neurons, increased global cortical thickness and larger total cortical area compared to women [89,90]. In addition, in a study of callosal thickness, it was found that the CC was thicker in men, but the sex difference was no longer found after scaling for total brain volume [91].

Biegon et al. [92] measured the cross-sectional area of the CC and splenium from patients with AD, age-matched elderly controls, and young controls. The midsagittal Magnetic Resonance Imaging (MRI) measurements showed a reduction in the callosal area with age in men, which was not observed in women. An early study revealed that males’ average axon diameter and total tract volume in the CC are more significant. Yet, females have a higher total number of axons in this tract [93]. Another study [94] analyzed the hippocampal volume of the healthy control population. The Alzheimer’s Disease Neuroimaging Initiative (ADNI) database showed that hippocampal volume (mean = 7175 ± 886 mm^3^, N = 187 women vs. mean = 7539 ± 935 mm^3^, N = 192 men) is slightly (5%) but significantly higher in elderly men compared to women, while the sex difference in ICV was 12.7% (mean = 1423 cm^3^ in women and mean = 1604 cm^3^ in men). Pruessner et al. [95] studied the hippocampal volume in early adulthood (39 men and 41 women, ages 18–42 years old) and discovered a significant negative correlation with age for both left and right hippocampus in men (r = −0.47 and −0.44) but not in women (r = 0.01 and 0.02). From 30 years onward, men’s hippocampal volume declines about 1.5% annually.

Furthermore, studies [96,97] have consistently shown that the left hemisphere auditory and language-related regions are proportionally expanded in women versus men, suggesting a possible structural basis for the widely replicated sex differences in language processing. At the same time, regarding men, the primary visual and visuospatial association areas of the parietal lobes were proportionally more extensive, in line with prior reports of relative strengths in visuospatial processing and skills. Martinez et al. [89] showed that the sex difference in regional brain volumes diminishes with age. Understanding sex differences in these areas and skills are of interest in the context of TBI since many resulting disorders affect language and spatial skills. Language and visuospatial skills are highly relevant for the early detection of TBI-induced pathologies. For example, American football-related concussions have received increasing attention due to neurological disorders seen among players, highlighting the need for a rapid screening tool. The King–Devick (KD) test requires eye movements, language function and attention to perform functions that reflect suboptimal brain function in concussion. The athletes are required to read a series of numbers on three test cards quickly and are judged according to their performance on the KD test [98]. Another example would be the in elderly widely used cognitive impairment screening, the Clock Drawing Test (CDT). The CDT is a valuable cognitive screening test for many cognitive functions, including selective and sustained attention, auditory comprehension, verbal working memory, numerical knowledge, visual memory and reconstruction, visuospatial abilities, and on-demand motor execution (praxis), as well as an executive function [99]. In previous research examining sex differences in CDT performance, mixed results were found, with some suggesting an influence of sex [100,101] and others finding no significant difference [102].

### 3.3. Sex Differences in Axonal Structure

A recent study used human and rat neurons to develop in vitro 2 mm long micropatterned axon tracts that were genetically either male or female [103]. The potential sex differences in axon structure and responses to Traumatic Axonal Injury (TAI) were examined in an ultrastructural analysis. Dollé et al. [103] showed for the first time that female axons were consistently smaller with fewer microtubules than male axons. Human male axons showed an approximately 80% increase in cross-sectional area (91.913 nm^2^ vs. 50.981 nm^2^) and a 55% rise in the number of microtubules per axon when compared to female axons. Numerical modelling of TAI revealed that these structural differences place female axonal microtubules at greater risk of failure under the same applied loads than male axons. Because of the smaller diameter and greater periphery to area ratio for smaller diameter axons and the resulting dominant peripheral forces, there were higher longitudinal strains along microtubules in smaller diameter axons with fewer microtubules than in larger diameter axons with more microtubules, leading to more significant mechanical failure of the microtubules. The in vitro model showed that dynamic strain-injury to axon tracts induced greater undulation formations resulting from the mechanical breaking of microtubules and more significant calcium influx shortly after the same level of injury in female axons. A day post-injury, female axons exhibited significantly more swellings and more substantial loss of calcium signalling function than male axons. In addition, female axons displayed more significant axon transport interruption and degeneration than male axons receiving the same injury.

### 3.4. Sex Effects on Cervical Spinal Cord Injury

The cervical spine region is particularly vulnerable to injury due to its natural proximity to the head, the high degree of freedom and the lack of protection relative to the other areas of the spine [104]. Cervical musculature is primarily responsible for maintaining posture and stabilizing the head [105]. Neck strength can reduce the risk for neck-related brain injuries in accidents, and sports [106]. Studies have consistently shown that neck strength is higher in men than in women [106,107] and it has been demonstrated that neck strength declines with age [108,109]. Although there has been much research on TBI and concussion, few studies assess the prevalence of comorbid (co-occurring) neck injuries, such as whiplash. Both the clinical presentation and the diagnostic hallmarks of concussion and whiplash have considerable overlap [110]. Furthermore, they have been found to co-occur commonly, which makes it, in particular, challenging for clinical differentiation [111]. The biological differences between the sexes might contribute to the partially observed increased vulnerability among females to sustain whiplash in motor vehicle collisions (MVC) [112,113], worse outcome after injury [114], and higher risk of concussion [106]. On the one hand, a recent study [115] showed that Canadian females between the ages of 5–49 with a concussion-related emergency department (ED) visit had a significantly higher rate of comorbid neck injury across all types of injuries. On the other hand earlier studies by Hasler et al. [116] and Fuji et al. [117] showed contradictory results.

### 3.5. Hormonal Differences between Sex in Context of HN Injury

After birth up until puberty, boys and girls experience a low level of sex hormones. After puberty, the predominant sex hormone in males is testosterone. Testosterone production declines with age [118]. In females, estrogen and progesterone are present cyclically until menopause. Nonetheless, both testosterone is present in females, as well as estrogen and progesterone being present in males [119].

Clevenger et al. [120] used a Controlled Cortical Impact (CCI) model of TBI in mice to test whether female mice would demonstrate less injury than male mice due to the protective role of endogenous steroids. The results indicated that female sex steroids indeed reduce brain sensitivity to TBI and that reduced neuroinflammation may play a role in the relative protection observed in females. Male and ovex (ovariectomized) female mice showed significant motor deficits and larger injury sizes than intact females.

Recent studies [38,121,122] have confirmed the close relationship between TBI and pituitary dysfunction. The pituitary gland helps control human growth, blood pressure, sex organs, and human metabolism. Around 30% of TBI long-term (12 months or more following trauma) survivors of hypogonadism deal with the effects of hypogonadism; it brings crucial implications for future studies regarding the effects of sex in TBI outcomes.

### 3.6. Non-Hormonal Factors

However, several studies have raised questions about the importance of female sex hormones following TBI. The fact that post-menopausal women had better outcomes compared to men [68,75], as well as poor results [123] of progesterone in phase III clinical trials question the importance of sex hormones in determining TBI outcome. Therefore, these findings support the hypothesis that factors beyond sex hormones are likely essential contributors to sex differences after TBI.

In contrast to the many studies focusing on the hormonal basis of sex differences in TBI, the apparent chromosomal differences were practically neglected. Alternatively, sex differences could arise from chromosomal differences wherein XX (female) or XY (male) complement genes drive brain development [124]. To balance gene expression, one of the X chromosomes undergoes inactivation in female embryos [125]. Since some genes escape the inactivation and remain transcriptionally active, they are abundant and have a higher gene expression compared to males [126,127]. In the context of TBI, this overexpression of genes is particularly interesting since the X chromosome is enriched in genes frequently expressed in neural tissue. For example, most escape genes identified in studies in mice contributed to neuronal differentiation, cell survival, dendritic outgrowth, and synaptic density [126,128].

In a study by Lentini et al. [129], hormonal and chromosomal influences on the brain were dissected by comparing XXY males (Klinefelter syndrome) with XY males and XX females. Results showed that there are indeed sex differences associated with X-chromosome load, such as cerebellar and precentral grey matter volumes. In contrast, sex differences in the parahippocampus, occipital cortex and amygdala were associated with testosterone levels. Since these regions of the brain are responsible for stress responses [130] and given the fact that post-TBI symptoms such as anxiety and depression are more likely to be found in women than men, while in men, amnesia and confusion are more often reported [131]; the anatomical differences in the amygdala, hippocampus and the prefrontal cortex might contribute to different post-TBI symptoms. Sex differences can be observed even on the structural level of axons. A study showed that axons from females were smaller and had fewer microtubules than males. In addition, a recent in vitro study of the female axons showed a greater swelling response and a more extensive loss of calcium signalling compared to males after a strain-induced deformation [103]. The sex differences in axon calibre and structure are likely due to the Y chromosome [103].

Another factor contributing to different TBI outcomes in sexes may be mitochondrial differences. Mitochondria are an essential organelle for most eukaryotic cells, especially neurons. Mitochondria are crucial for regulating calcium homeostasis, developmental and synaptic plasticity, neurotransmitter synthesis, free radical production, and apoptosis in neurons and glia [132]. Studies point to the sex-based disparity in neuronal metabolism. While the difference in processes in males and females has been shown under physiological conditions, it appears to be magnified under pathophysiological conditions [133]. Given mitochondrial dysfunction and the resulting bioenergetic disruption are fundamental to the injury cascade of TBI. Since mitochondria have marked sex differences, this is an area that warrants further investigation [19].

It has been recognized that estrogen and progesterone regulate oxidative metabolism in brain mitochondria. These steroids can induce alterations in the central nervous system by supporting balanced and efficient bioenergetics, reducing oxidative stress and attenuating endogenous oxidative damage [134].

### 3.7. Sex and Age Differences in Brain Swelling

The high incidence of idiopathic intracranial hypertension in premenopausal women (<50 years of age) [135,136] and the known effects of female gonadal hormones on the bodies fluid balance [137] support the likelihood of a sex difference of brain swelling incidence and intracranial hypertension following TBI, especially in premenopausal women. Studies have consistently shown that brain swelling and increased intracranial pressure are risk factors for poor outcomes in animal models and in human TBI [138,139,140].

Another study that supports sex and age differences in TBI outcome [141], has shown that female patients had a significantly greater frequency of brain swelling and intracranial hypertension compared with male patients (35% compared with 24% [*p* < 0.0008] and 39% compared with 31% [*p* < 0.03], respectively). The most significant difference was found in patients younger than 50 years. Female patients younger than 51 years old showed the highest rate of brain swelling and intracranial hypertension (38% compared with 24% [*p* < 0.002] and 40% compared with 30% [*p* < 0.02], respectively, when compared to male patients younger than 51 years of age). Thus, premenopausal females may benefit from more aggressive treatment and monitoring of intracranial hypertension after TBI.

## 4. Sex-Specific Numerical Approaches on HN Injuries Prediction

For several years, cadavers, animals and sometimes volunteer living subjects have been used to provide valuable information regarding HN injuries. However, this kind of experimentation is sometimes denied by health committees due to obvious ethical and moral issues.

Alternative scientific community approaches include using test dummies to gather impact data (usually from motor vehicle crashes) and results. Some examples of these dummies include the famous Hybrids (for frontal impact), the SIDs (for side impact), the BioRID (for rear impacts), the CRABI (a child dummy), and more advanced models such as THOR (advanced male dummy) [142]. These dummies are usually of a standardized 50th percentile male. Even though some female crash test dummies exist [143], they are not mandatory to use in most vehicle crash tests; this correlates with a much higher percentage of belt-restrained female drivers (≈47%) likely to sustain severe injuries when compared to belt-restrained male drivers [144,145].

Despite being invaluable for several applications, crash test dummies present numerous disadvantages, such as replacing parts after a crash test and incurring high costs to the company performing such tests. In addition, several biomechanical factors of the different components of the human HN are impossible to simulate using a dummy. For these reasons, the introduction of Finite Element (FE) modelling of the human HN allows accurate estimation of the same biomechanical responses.

The Finite Element Method (FEM) is a known method of solving differential equations by discretizing a continuous physical domain into finite elements [146]. In this case, the HN structures can be modelled with finite elements and, with the appropriate material properties, boundary conditions and simplifications, determine how a specific impact or acceleration-deceleration can affect these structures.

### 4.1. Finite Element Head Models

Over the years, several Finite Element Head Models (FEHMs) have been developed, starting as two-dimensional plane deformation models [147,148,149,150]. From this period on, technological advancements allowed modelling of more detailed FEHMs, such as the introduction of 3D modelling, the capability of mesh refinement and simulation of non-linearities such as plasticity or hyperelasticity. Table 1 displays a literature review on prominent FEHMs throughout the years, adapted from Tse et al. [142], revised and completed with more recent advancements in FEHMs, some listed by McGill et al. [151].

These models are often validated using experimental data obtained with cadaveric specimens subject to blunt impacts or induced accelerations. The first relevant experiments were performed by Nahum et al. [152,153], recording translational acceleration-time and intracranial pressure (ICP)-time histories using biaxial accelerometers fixed to the skull. Experiment #37 of a 42-year-old male is reported in detail, including the plot of each response metric throughout the impact, impact conditions, and peak pressure responses. Consequently, this was the most commonly replicated test in FEHM validation for several years. Nahum et al. [152,153] performed impact tests on both male and female cadaveric subjects; from the tests, experiment #37 (male subject) has sample data records of pressure-time responses in different intracranial locations, and for this reason, is widely regarded as the validator for FEHMs.

Two decades after, in 1992, Trosseille et al. [154] investigated factors influencing the ICP response. This experiment’s main objective was to develop a tool to validate FEHM for automotive crash research. Specimens contained pressure transducers inserted in the arachnoid space on the frontal, parietal and occipital regions and in the third and lateral ventricles. Samples also contained accelerometers in the brain tissue in four different locations to compare the relative motion of the structure. This investigation used unspecified-sex subjects.

At this point in time, after Trosseile et al. [154] experiments, studies with relative brain motion started to be introduced, for providing a much more accurate validation of FEHMs when compared to intracranial pressure data.

Another main experiment used to validate FEHMs are Hardy et al. studies [155,156]. This revolutionary study measured relative brain motion concerning the skull using a high-speed biplanar X-ray technology to track target points on cadavers during a total of 45 impacts at velocities ranging from 2.5 to 3.9 m/s. The specimens were also fitted with accelerometers in the skull to record the kinematic response of each impact. The target points detected by the X-ray are named Neutral Density Targets (NDT), tin granules encased in polystyrene capsules with a similar density to the surrounding tissue on the site of implantation in the brain. The main difference between both experiments was the location of the NDT in the brain. In the 2001 investigation [155] the NDT were arranged in in columns, while in the 2007 study [156] the authors arranged the NDT in clusters of seven, creating an array of triads about a central NDT, with an approximate radius of 10 mm. Hardy et al. [156] performed studies on male and female subjects, the recording head and brain responses for each specimen. This experiment poses a reasonable sex-specific approach to female FEHM validation and is currently the most commonly replicated test to validate FEHMs.

The latest experiments to be implemented in FEHM validation are Alshareef et al. [157,158]’s sonomicrometry studies, being used as an alternative to biplanar X-ray technology using NDT. This new method used sonomicrometry crystals to quantify brain deformation with respect to time, in response to dynamic rotation pulsesapplied to the cadaveric head. The crystals can transmit and receive ultrasound pulses and calculate distance at a high frequency using the speed of sound of the tissue [157]. In the 2018 study [157], the crystals initial testing was performed in situ with porcine brain tissue to test the implantation technique, data recording and possible brain tissue damage. A total of 24 crystals were inserted into the brain tissue (receiving crystals), and eight were affixed to the inner skull (transmitting crystals). Alshareef et al. [158]’s 2020 investigation employed a similar methodology using 30 sonomicrometry transmitters in the brain and six receivers on the skulls of six cadavers. This work has been implemented to investigate the advancement and application capabilities of the Global Human Body Models Consortium (GHBMC) FEHM [159,160]. The 2018 study [157] uses a 53-year-old male specimen. The 2020 study used six HN specimens, four of which were female. However, the analysis focused on the relationship between the kinematics of the impulse created by the Rotational Test Device (RTD) and the brain’s physical response; consequently, the data provided is of limited potential for FEHM validation [151].

Overall, sonomicrometry allows the relative displacement to be represented along all three anatomical axes. In contrast, the biplanar X-ray technique is limited to two directions since targets cannot be placed at multiple depths, since it would cause optical interference, restricting the placement of NDT to planar columns or regional clusters. These limitations also require that different NDT patterns be used for different tests, denying having multiple experiments with all three anatomical axis data.

The novelty of Alshareef et al. [157,158]’s approach shows promise for FEHM validation. However, for proper validation of a female FEHM, sex-specific brain deformation data would provide a more accurate alternative to using male or unspecified-sex experimental data.

**Table 1:** Literature review on prominent Finite Element head models.

Authors	Year	Type	Model Description	Validation
Kenner and Goldsmith [161]	1972	3D	Compressible fluid in a spherical shell (with an elastic skull shell and a viscoelastic brain fluid).	
Hardy and Marcal [147]	1973	2D	Linear elastic isotropic skull.	
Nickell and Marcal [148]	1974	2D	Linear elastic skull used for a vibration response study.	
Chan [162]	1974	3D	Linear viscoelastic head bonded to a linear viscoelastic spherical shell and a prolate ellipsoid.	
Shugar [163]	1975	3D	Three-layered skull with brain matter, modelled as a nearly incompressible material.	
Shugar and Katona [164]	1975	3D	Thin layer replicating the sub-arachnoid space (that houses the CSF).	
Ward and Thompson [165]	1975	3D	Rigid skull with CSF and a linear elastic core.	
Khalil and Hubbard [166]	1977	3D	Single or multi layer circular and ellipsoidal shells with a fluid-filled cavity (elastic scalp and skull layers and viscoelastic brain fluid).	
Nahum et al. [153]	1977	3D	Linear elastic brain	Pressure
Hosey and Liu [167]	1982	3D	Homeomorphic HN model with skull and brain (also including falx, dura mater, scalp and CSF) and cervical spinal cord and column.	Initial inertial characteristics of the brain
Ueno et al. [149,150]	1989	2D	two-dimensional model with a rigid skull and a linear elastic brain	Pressure
	1991			
Ruan et al. [168,169]	1993	3D	Layered skull, cerebral spinal fluid and brain modelled as brick elements with reduced integration. The thin elements such as dura mater, scalp and falx cerebri were modelled as membrane elements. Developed the WSUBIM version I, which including the scalp, a three-layer skull, dura mater, falx cerebri, brain and CSF.	Pressure (with Nahum et al. [153]’s frontal scenarios)
	1994			
Zhou et al. [170]	1995	3D	Improved the WSUBIM version I, refining the mesh.	Pressure (with Nahum et al. [153]’s frontal scenarios); Relative brain motion magnitude
Kumaresan and Radhakrishnan [171]	1996	3D	Homeomorphic FEHM including skull, CSF, brain (with arachnoid, pia and dura mater) and neck.	
Kang et al. [172,173]	1997	3D	Developed the SUFEHM model.	Pressure (with Nahum et al. [153]’s and Trosseille et al. [154]’s frontal scenarios); Stress tests from motorcycle accident
	1999			
Zhang et al. [174]	2001	3D	Developed WSUBIM version II, with improved facial characteristics and introducing a sliding interface between the skull and brain.	Pressure (with Nahum et al. [153]’s frontal scenarios)
Kleiven and Hardy [175]	2002	3D	Developed the KTH-FEHM, consisting of scalp, skull, brain, meninges, CSF, bridging veins and a neck. Also included a sliding boundary condition between the skull and brain.	Pressure (with Nahum et al. [153]’s and Trosseille et al. [154]’s frontal scenarios); Relative brain motion (Hardy et al., 2001 [155])
King et al. [176]	2003	3D	Final version of the WSUBIM with a viscoelastic brain and elastic-plastic skull.	
Horgan and Gilchrist [177]	2003	3D	Developed the UCDBTM version I, including a scalp, skull, dura, all the main brain components and CSF.	Pressure (with Nahum et al. [153]’s frontal scenarios)
Takhounts et al. [178,179]	2003	3D	A fast computation model (SIMon FEHM), that didn’t include both cerebellum and midbrain.	Pressure (with Nahum et al. [153]’s and Trosseille et al. [154]’s frontal scenarios); Relative brain motion (Hardy et al. [155])
	2008		Advanced model with skull, dura, CSF and brain.	
Belingardi et al. [180]	2005	3D	FEHM generated from CT and MRI data, which included the scalp, skull with facial bones, dura, CSF, brain, falx and tentorium.	Pressure (with Nahum et al. [153]’s frontal scenarios)
Zong et al. [181]	2006	3D	Simplified model with a 3-layer skull, incompressible CSF and brain.	Pressure (with Nahum et al. [153]’s and Trosseille et al. [154]’s frontal scenarios)
McAllister et al. [182]	2012	3D	Developed a model for sports-related concussion, the Dartmouth Subject-Specific Finite Element Head Model (DSS FEHM), using a MRI-segmented brain, falx and skull.	Relative brain motion (Hardy et al. [156])
Mao et al. [183]	2013	3D	Developed the Global Human Body Models Consortium (GHBMC) FEHM version I. Using CT and MRI data, a high-quality, extensively validated FEHM composed of cerebrum, cerebellum, brainstem, CC, ventricles and thalamus.	Pressure (with Nahum et al. [153]’s and Trosseille et al. [154]’s frontal scenarios); Relative brain motion (Hardy et al. [155,156])
Yang et al. [184]	2014	3D	FEHM developed for TBI prediction during vehicle collisions, using CT and MRI data. CSF is simulated as a fluid-filled cavity.	Pressure (with Nahum et al. [153]’s and Trosseille et al. [154]’s frontal scenarios); Relative brain motion (Hardy et al. [155])
Sahoo et al. [185]	2014	3D	Improvement on the SUFEHM with the implementation of FA, axonal fiber orientations using diffusion tensor imaging (DTI) and visco-hyperelastic brain material constitutive laws.	Pressure (with Nahum et al. [153]’s frontal scenarios); Relative brain motion (Hardy et al. [155,156])
Ji et al. [186]	2015	3D	Developed the DHIM including the cerebrum, cerebellum, brainstem, CC, CSF, pia, dura, tentorium, falx, diploe, foramen magnum, cortical bones and scalp.	Pressure (with Nahum et al. [153]’s and Trosseille et al. [154]’s frontal scenarios); Relative brain motion (Hardy et al. [155,156])
Zhao et al. [187]	2015			
Atsumi et al. [188]	2016	3D	Parametric FEHM created for the determination of factors causing brain tissue displacements and ICP in head impacts. Composed of cerebrum, Skull, CSF, cerebellum, falx, pia and superior sagittal sinus.	Pressure (with Nahum et al. [153]’s and Trosseille et al. [154]’s frontal scenarios); Relative brain motion (Hardy et al. [155])
Miller et al. [189]	2016	3D	Developed the ABM, the first dynamic FEHM to include 3D gyri to study detailed brain deformations.	Relative brain motion (Hardy et al. [155,156])
Miyazaki et al. [190]	2017	3D	Creation of a FEHM to correlate brain node motion with an anthropometric test device (ATD) head mounted on an AM50 Hybrid III dummy.	Relative brain motion (Hardy et al. [156])
Toma et al. [191,192,193]	2018	3D	Developed the first Fluid–Structure Interaction (FSI) model, capable of simulating the CSF flow around the brain.	Pressure (with Nahum et al. [153]’s frontal scenarios)
	2020			
Fernandes et al. [194] Migueis et al. [195] Barbosa et al. [196] Costa et al. [197]	2018–2020	3D	Developed Yet Another Head Model (YEAHM), a geometrically detailed finite element brain model, with a detailed sulci and gyri modelling. The skull model was later segmented with sutures, diploë and cortical bone and later completed with bridging veins (BV) to predict subdural haematoma.	Pressure (with Nahum et al. [153]’s frontal scenarios); Skull fracture prediction (Huang et al. [198]’s experiment); Subdural haematoma prediction (Depreitere et al. [199]’s experiment)
Wu et al. [159]	2019	3D	Developed the Global Human Body Models Consortium (GHBMC) FEHM version II. Embedded to the base model WM fibre tracts using 1D cable elements with hyper-viscoelastic constitutive models.	Relative brain motion (Hardy et al. [155,156]); Brain deformation (Alshareef et al. [157])
Khanuja and Unni [200]	2020	3D	High-quality, comprehensive FEHM with detailed cerebral sulci and gyri structures. Composed of skull, CSF, cerebrum, cerebellum, and brainstem.	Pressure (with Nahum et al. [153]’s and Trosseille et al. [154]’s frontal scenarios)
Hassan et al. [201]	2020	3D	Developed a simplified FEHM with low computational cost.	Pressure (with Nahum et al. [153]’s frontal scenarios)
Trotta et al. [202]	2020	3D	Developed the UCDBTM version II with updated mechanical properties and a low friction coefficient between the skull and the scalp.	Relative brain motion (Hardy et al. [155,156])
Li et al. [203]	2021	3D	Developed the ADAPT model, an anatomically detailed FEHM with conforming hexahedral meshes, with WM fiber tracts. This model also includes a mesh-morphing approach for subject-specific modelling.	Pressure (with Nahum et al. [153]’s frontal scenarios); Relative brain motion (Hardy et al. [156])

Aside from the models displayed in Table 1, some versions of open-source head and full body models are also available for researchers that do not intend to develop their own FEHM, such as the Total Human Model for Safety (THUMS) and GHBMC models.

### 4.2. Finite Element Neck Models

While FEHM are more commonly found in the literature, several Finite Element Neck Models (FENM) have been developed to study the different types of WAD, the term given for the collection of symptoms affecting the neck that are triggered by an acceleration-deceleration mechanism, usually associated with motor vehicle crashes [204].

Several studies report sexual dimorphism of cervical anthropometry. Vasavada et al. [205] reported that female vertebrae from C3 to C7 were significantly smaller than male vertebrae in the anterior-posterior dimension. The medial-lateral dimension did not follow the same downscaling. The study concluded that male and female necks are not geometrically similar and indicated that a female-specific model is necessary to study sex differences in neck-related disorders. Stemper et al. [206] found that linear and areal dimensions of the cervical spine were greater for male volunteers, indicating a more stable spinal column that may be more capable of resisting inertial loads applied during automotive rear impacts. The study also demonstrated the fundamental difference in male and female spinal geometry that cannot be accounted for by simply scaling anatomical dimensions. The similar conclusions of both studies suggest the need to consider sex-specific cervical spine anatomy. Additionally, females also tend to have greater ligamentous laxity [207,208,209,210] and smaller neck muscles in absolute and relative terms [105,107,211,212,213].

All these findings suggest the need for sex-specific FENM with muscle integration to accurately predict the motion of the HN system and the resulting outcome of a particular impact or acceleration-deceleration scenario.

Over the years, several FENMs have been developed, starting from two-dimensional structures [214] and evolving to three-dimensional models with subject-specific anthropometry, properly validated with experimental intervertebral motion data. Table 2 contains a literature review of the most prominent FENM.

The first relevant experiment used to validate FENMs was conducted by Moroney et al. [217], measuring the load-displacement behaviour of 35 cervical spine segments, namely compression, shear, flexion, extension, lateral bending, and axial torsion. For load-displacement testing, each motion segment was mounted so that the inferior vertebra was rigidly attached to the base of a testing apparatus while the superior vertebra was free to move in response to the loads applied. The measurements were performed using six dial gauges aligned with a motion segment reference plane to allow the measurement of all the six components of segment motion. Three years later, in 1991, Traynelis et al. [224] measured six modes of angular motion (flexion, extension, right and left lateral bending, and right and left rotation) with a testing apparatus similar to Moroney et al. [217]. The measurements were performed using Light-Emitting Diodes (LED), rigidly attached to each vertebra (from C3 to C7), and monitoring the LED motion with a photoelectric system. Panjabi et al. [220,221] measured multidirectional intervertebral motions (flexion, extension, axial rotation and lateral bending) by generating pure moments using a pulley system mounted on top of the specimen. The measurements were performed using a stereophotogrammetry system, applying markers to the anterior aspect of each vertebral body and the occiput. Finally, Yoganandan et al. [239,240] determined the coronal and axial moment-rotation responses of the cervicothoracic spinal columns under the lateral bending mode. The rotational kinematics in the coronal and axial planes were obtained using retroreflective targets in each vertebra. From all these validation experiments, some utilized male and female cervical spinal cords [239,240] however, none conducted sex-specific data analysis, and the results were generalized for both sexes. This generalization hinders the successful validation of both female and male neck models since cervical anthropometry is sex-specific [205,206].

The only authors to conduct sex-specific intervertebral motion experiments were Nightingale et al. [227,228], having performed two individual researches for male [228] and female [227] specimens, and Stemper et al. [235,236,237]. These experiments were used to validate the FENM developed by Osth et al. [232] which later incorporated a skull and soft tissues [233]. This was the first and only FENM found in the literature that was developed using a female CT scan and validated using female intervertebral motion experiments. The 2016 work [232] was the steppingstone for female FENM, and the 2017 work [232] was the first attempt to develop a full female HN system. While the authors’ model was designed to develop automotive protective systems addressing WAD, future model improvements could include brain structures to study TBI mechanisms.

## 5. Discussion

As noticeable, this work overlaps the biological aftermath of an inertial loading scenario with the numerical prediction models created throughout the years. The apparent gap between the two is clear although, in the authors’ perspective, the future of this field is the transition to the simulation of loading scenarios with the inclusion of biological responses that are triggered after an impact, instead of a pure mechanical analysis on the effects in soft tissue.

To note that there are multiple considerations to take in account when evaluating an impact and its aftermath, such as loading environment, age of the subject that may display different psychiatric phenotypes after TBI, tissue response (depending on the strain-rate of the tissue), physiological influences (intracranial pressure, pulse), muscle contractions, among several others.

The topic of subject variability is also highlighted, that does not purely depend on the sex of the subject, but also its general anthropometry and physical condition, and that question also raises the discussion of how much detail should a numerical model also possess. By trying to achieve subject-specific detailed structures, one also deviates from trying to create a model that biofidelically represents the average response of the average subject.

Even if utopically one represents every major anthropometric group of the population, the validation of those models would be even more challenging. How does one obtain the anthropometrically perfect cadaveric subject to validate each model created?—When straying from the most generic anthropometric model to a detailed subject-specific model, the validation used is exponentially more important, since a misrepresentation of the biomechanical motion of that brain would compromise the whole purpose of having a subject-specific model.

Regarding neck injuries, this topic is better explored, since the result of an injury is usually well-correlated with the mechanical conditions of that impact. Taking in account the musculature of the subject, anthropometry and acceleration scenario, it is possible to predict the outcome on the patient, if the correct boundary conditions are implemented in the model. On that topic, research has been performed using female anthropometry which, as discussed previously, does affect the injury outcome.

## 6. Wrap-Up and Future Directions

The present work aimed to establish a survey on the most important works dealing with head-neck injuries and to which extent the sex variability is being—or not—taken into account. The pioneers working in the field decades ago focused primarily on the average male patient. Fortunately, society as a whole evolved to recognise the critical differences between sexes regarding traumatic brain and neck injuries in terms of immediate and long-term responses and sequelae. From the substantial number of references herein presented and described with the possible detail of a state-of-the-art paper, it is undeniable that such distinctions exist and shall not be ignored. This topic is not consensual [241]; fruitful discussions are expected—and encouraged—in the years to come. As Giudice [242] points out, one should not disregard the intrinsic variability of the brain’s structure and functions within the same sex. Some characteristics might even overlap for different sexes. Individual variability is definitely the most challenging task for biomechanics researchers trying to establish a pattern of predictability in terms of the body—and particularly the head-neck system—response under mechanical loading.

Overall, sexual differences must gather the same level of attention regarding variability as other parameters such as age or clinical history. In doing so, future research lines on this must avoid radical generalizations (such as neglecting the differences between males and females entirely) and also avoid creating rigid subdivisions between sexual characteristics, allowing what can be called overlapping areas in terms of study.

Therefore, future research trends on this topic are expected to include the development of computational models, such as finite element ones, capable of parametrizing the differences between and within sexes by computing results dependent on the individual specific characteristics. This methodology would make possible more advances in medicine by giving deeper insights into injuries evaluation, in law to help solve cases of domestic abuse and in engineering to develop, for instance, specific protective equipment or safety systems that are safe for both males and females.

## Figures and Tables

**Table 2:** Literature review on prominent Finite Element neck models.

Authors	Year	Type	Model Description	Validation
Saito et al. [214]	1991	2D	Two triangular mesh models, a normal and a post-laminectomy model to compare the differences leading to post-laminectomy syndrome.	
Maurel et al. [215]	1997	3D	Parameterized FENM, including the complete lower cervical spine, allowing the model to fit different morphologies of vertebrae.	Axial torque, lateral flexion, flexion and extension (Moroney [216], Moroney et al. [217], Pelker et al. [218])
Zhang et al. [219]	2006	3D	Developed a geometrically accurate, nonlinear C0–C7 cervical spine model, based on the geometry of a human cadaver specimen.	Axial torque, lateral flexion, flexion and extension (Panjabi et al. [220,221])
Kallemeyn et al. [222,223]	2009	3D	Development of a functional spinal unit obtained using a CT scan and meshed using the multi-block technique. The model consisted only of hexahedral elements.	Axial torque, lateral flexion, flexion and extension (Moroney et al. [217], Traynelis et al. [224])
	2010	3D	Development of a cervical spine model using the multi-block technique. The model was divided to allow individual testing.	In-house experimental motion data.
Panzer et al. [225]	2011	3D	Developed a detailed cervical spine finite for the evaluation of global kinematics and tissue-level response.	Axial torque, lateral flexion, flexion and extension (Wheeldon et al. [226], Nightingale et al. [227,228], Dibb et al. [229])
Toosizadeh and Haghpanahi [230]	2011	3D	Geometrically accurate, non-linear model of C0–C7, using CT scan data.	Axial torque, lateral flexion, flexion and extension (Wheeldon et al. [226], Nightingale et al. [228])
Erbulut et al. [231]	2014	3D	Asymmetrical full cervical spine model to investigate the influences of ligaments, facet joints, and disk nucleus on the stability of the model during flexion and extension.	Axial torque, lateral flexion, flexion and extension (Traynelis et al. [224], Panjabi et al. [221], Wheeldon et al. [226], Nightingale et al. [227,228])
Òsth et al. [232,233]	2016	3D	Developed a ligamentous cervical spine of a female subject intended for biomechanical research on the effect of automotive impacts.	Axial torque, lateral flexion, flexion and extension (Panjabi et al. [221,234], Nightingale et al. [227])
	2017	3D	Used the previously created ligamentous cervical spine and incorporated a skull and soft tissues.	Rear impact experiments from Stemper et al. [235,236,237]
Cai et al. [238]	2020	3D	Developed a model of the cervical spine (C3–C7), with six degenerative models simulating mild, moderate, and severe grades of disc degeneration at C5–C6, using CT scan data.	Axial torque, lateral flexion, flexion and extension (Traynelis et al. [224], Panjabi et al. [221], Wheeldon et al. [226], Yoganandan et al. [239,240])

## Data Availability

Not applicable.
